# Development and validation a simple scoring system to identify malignant pericardial effusion

**DOI:** 10.3389/fonc.2022.1012664

**Published:** 2022-12-01

**Authors:** Xiaxia Jin, Lingling Hu, Meidan Fang, Qiaofei Zheng, Yuan Yuan, Guoguang Lu, Tao Li

**Affiliations:** ^1^ Department of Clinical Laboratory, Taizhou Hospital of Zhejiang Province, Taizhou Enze Medical Center (Group), Linhai, Zhejiang, China; ^2^ Department of Cardiovascular medicine, Taizhou Hospital of Zhejiang Province, Taizhou Enze Medical Center (Group), Linhai, Zhejiang, China

**Keywords:** malignant pericardial effusion, diagnosis, nomogram, scoring system, atypical cell

## Abstract

**Background:**

Malignant pericardial effusion (MPE) is a serious complication in patients with advanced malignant tumors, which indicates a poor prognosis. However, its clinical manifestations lack specificity, making it challenging to distinguish MPE from benign pericardial effusion (BPE). The aim of this study was to develop and validate a scoring system based on a nomogram to discriminate MPE from BPE through easy-to-obtain clinical parameters.

**Methods:**

In this study, the patients with pericardial effusion who underwent diagnostic pericardiocentesis in Taizhou Hospital of Zhejiang Province from February 2013 to December 2021 were retrospectively analyzed. The eligible patients were divided into a training group (n = 161) and a validation group (n = 66) according to the admission time. The nomogram model was established using the meaningful indicators screened by the least absolute shrinkage and selection operator (LASSO) and multivariate logistic regression. Then, a new scoring system was constructed based on this nomogram model.

**Results:**

The new scoring system included loss of weight (3 points), no fever (4 points), mediastinal lymph node enlargement (2 points), pleural effusion (6 points), effusion adenosine deaminase (ADA≦18U/L) (5 points), effusion lactate dehydrogenase (LDH>1033U/L) (7 points), and effusion carcinoembryonic antigen (CEA>4.9g/mL) (10 points). With the optimal cut-off value was 16 points, the area under the curve (AUC), specificity, sensitivity, positive predictive value (PPV), negative predictive value (NPV), positive likelihood ratio (PLR), negative likelihood ratio (NLR) for identifying MPE were 0.974, 95.1%, 91.0%, 85.6%, 96.8%, 10.56 and 0.05, respectively, in the training set and 0.950, 83.3%, 95.2%, 90.9%, 90.9%, 17.50, and 0.18, respectively, in the validation set. The scoring system also showed good diagnostic accuracy in differentiating MPE caused by lung cancer from tuberculous pericardial effusion (TPE) and MPE including atypical cell from BPE.

**Conclusion:**

The new scoring system based on seven easily available variables has good diagnostic value in distinguishing MPE from BPE.

## Introduction

Pericardial effusion (PE) is a common clinical syndrome that usually caused by infection, iatrogenic, and connective tissue diseases. Malignant pericardial effusion is the result of cancer invading the pericardium. Secondary involvement of pericardium is much more common than primary cardiac malignancies, and the most common causes include lung cancer, breast cancer, malignant melanoma, lymphoma, and leukemia (1). The incidence rate of pericardial involvement in malignant tumors is about 10-20% in all cancer patients. The incidence of MPE may increase with the increase of global cancer incidence rate and the overall survival rate (2, 3). MPE which indicates a poor prognosis is a serious complication of patients with advanced malignant tumors (4). The misdiagnosed and delayed treatment will directly lead to increased mortality. Therefore, rapid and accurate identification of MPE is not only the basis of diagnosis but also very important to inpatient treatment.

At present, pericardial biopsy and pericardiocentesis are still the main means to diagnose MPE. However, the pericardial biopsy is not easy to accept because of its invasiveness and potential complications. The cytological analysis is the gold standard for diagnosing MPE. But the evaluation of results largely depends on the professional knowledge of pathologists. The sensitivity is only 30-50%, and a large number of samples are required (5, 6). Moreover, it is difficult to diagnose some atypical cells only with cytological specimens. With the help of some auxiliary tools, this gray area can be reduced, which is helpful to achieve a clear diagnosis (7). Previous studies have shown that some laboratory indicators, such as various tumor markers, vascular endothelial growth factor, and serum BNP (8–10), have certain value in the differential diagnosis of benign and malignant PE. But the diagnostic accuracy still needs to be improved, and some new markers have not been widely used in clinical practice. Therefore, it is very important to design a simple, economical, and less traumatic method to identify MPE.

In this study, we conducted a retrospective study to develop a nomogram model based on clinical features and laboratory data to identify MPE from BPE. Next, we aimed to develop a new scoring system based on this nomogram for clinical practical application.

## Materials and methods

### Patient selection

We retrospectively analyzed the data of patients with pericardial effusion who underwent diagnostic pericardiocentesis in Taizhou Hospital of Zhejiang Province from February 2013 to December 2021. All patients met the following criteria: (1) PE confirmed by chest X-ray, CT, or ultrasound; (2) Patients undergoing diagnostic pericardiocentesis. Exclusion criteria: (1) patients with pericardial effusion of unknown etiology; (2) Patients with more than 30% missing information.

This study was approved by the ethics committee of Taizhou hospital, Zhejiang Province (K20220104). Informed consent was abandoned because it was a retrospective study.

### Data collection

Relevant data of the selected patients were collected, including: (1) clinical information, including gender, age, chest distress, chest pain, anepithymia, loss of weight, fever (fever is defined as body temperature > 37.5°C), heart rate, pleural effusion, size of effusion, pericardial hematocele, mediastinal lymph node enlargement; (2) blood laboratory data include high sensitivity C-reactive protein (hs CRP), total protein (TP), albumin (ALB), ADA, glucose (GLU), LDH, erythrocyte sedimentation rate (ESR), CEA; (3) Laboratory data of pericardial effusion include karyocyte count, effusion TP, effusion ALB, effusion ADA, effusion GLU, effusion LDH and effusion CEA. All numerical variables were converted into categorical variables according to the cut-off value which were obtained using receiver operating characteristic (ROC) curves (**Supplementary Figure 1**).

### Diagnostic criteria

MPE is diagnosed if the patient meets at least one of the following criteria: (1) tumor cells are detected in pericardial effusion cytology or pericardial biopsy; (2) proof of primary tumor; (3) atypical cells are detected in pericardial effusion and there is clinical evidence of tumor spread and exclusion of other potential causes of pericardial effusion (1, 11).

BPE is diagnosed if the patient meets at least one of the following criteria: (1) no cancer cells are detected in pericardial effusion cytology or pleural biopsy; (2) PE disappeared after etiological treatment and thoracic puncture. All BPE was confirmed by clinical and laboratory examination without any tumor signs.

Tuberculous pericardial effusion (TPE) is diagnosed if the patient meets at least one of the following criteria: (1) pericardial effusion or biopsy smear shows acid fast bacilli, or Mycobacterium tuberculosis culture or polymerase chain reaction is positive in other clinical samples; (2) Granulomatous inflammation in pericardial biopsy specimens; (3) After empirical antituberculosis treatment, the clinical manifestations and imaging findings of pericardial effusion were resolved.

### Statistical analysis

All data were statistically analyzed using R (Version: 4.0.5). Chi square test or Fisher’s exact test and lasso regression were used to screen the risk factors of MPE in the training set. Odds ratios (ORs) and their 95% confidence intervals (CIs) were estimated. A nomogram was developed to present the model according to the independent risk factors selected by multivariate logistic regression. The prediction accuracy of the model was evaluated by ROC curve AUC, calibration curve, decision curve analysis (DCA), and clinical impact curve (CIC). In order to make the prediction model more suitable for doctors’ use in clinical work, we modified the nomogram to the scoring system, and evaluated the diagnostic accuracy of this scoring system using AUC, specificity, sensitivity, PPV, NPV, PLR, and NLR in the training set and verification set. P<0.05 was considered to be statistically significant.

## Results

### Patient characteristics

**Figure 1:**
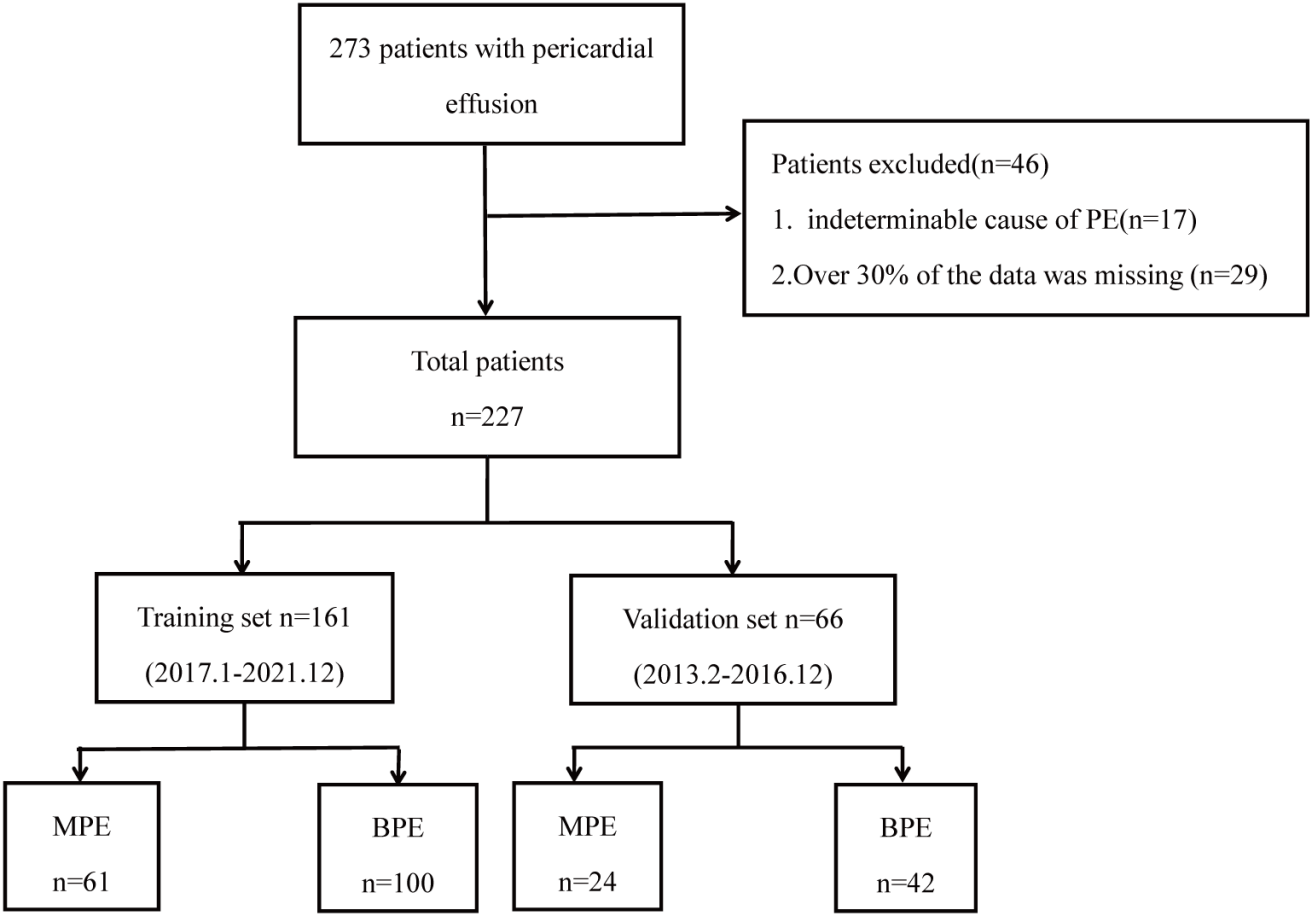
Flow chart of participant selection.

A total of 273 patients were diagnosed with pericardial effusion. 46 patients were excluded because they did not meet the inclusion criteria. A total of 227 patients (85 MPE and 142 BPE) were included in this study. The patient selection flow chart is shown in **Figure 1**. In 85 cases of MPE, tumor cells were found in 57 cases, atypical cells were found in 28 cases, and tumor cells or atypical cells were not found in 142 cases of BPE. The etiological classification of included patients is shown in **Table 1**. 85 patients with MPE and 142 patients with BPE were divided into a training group (admission in 2017 and later) and a validation group (admission before 2017).

**Table 1:** Baseline characteristics of the study set.

Paremeters	Training set (n = 161)	Validation set (n = 66)
Age (years)	67.0 (59.0, 76.0)	60.0 (49.2, 72.0)
Gender (%)		
Male	99 (61.5)	21 (47.0)
Female	62 (38.5)	35 (53.0)
Malignant pleural effusion (%)		
Lung cancer	46 (75.4)	18 (75.0)
Esophageal carcinoma	2 (3.3)	0 (0.0)
Breast carcinoma	5 (8.2)	3 (12.5)
Gastrointestinal carcinoma	4 (6.6)	1 (4.2)
Non-Hodgkin lymphoma	2 (3.3)	1 (4.2)
Cancer of unknown primary	2 (3.3)	1 (4.2)
Benign pleural effusion (%)		
Tuberculosis	26(26.0)	15 (35.7)
Cardiac injury syndromes	5 (5.0)	1 (2.4)
Autoimmune	5 (5.0)	2 (4.8)
Congestive heart failure	19 (19.0)	5 (11.9)
Cirrhosis	3 (3.0)	1 (2.4)
Nephrotic syndrome	8 (8.0)	1 (2.4)
Traumatic	3 (3.0)	1 (2.4)
Hypothyroidism	4 (4.0)	1 (2.4)
Infectious	6 (6.0)	2 (4.8)
Idiopathic	21 (21.0)	13 (31.0)

### Development and validation of nomogram and new scoring system

**Figure 2:**
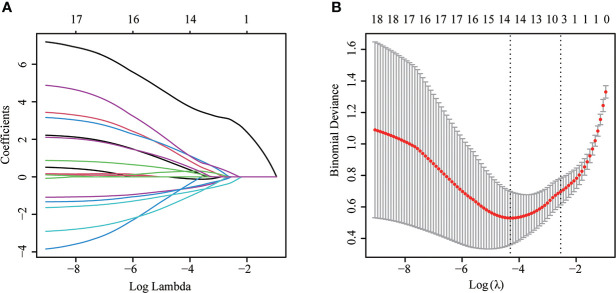
The LASSO logistic proportional hazard regression to screen risk factors for MPE. **(A)** The plot of partial likelihood deviance of LASSO logistic regression. **(B)** Plot of LASSO coefficient profiles.

**Figure 3:**
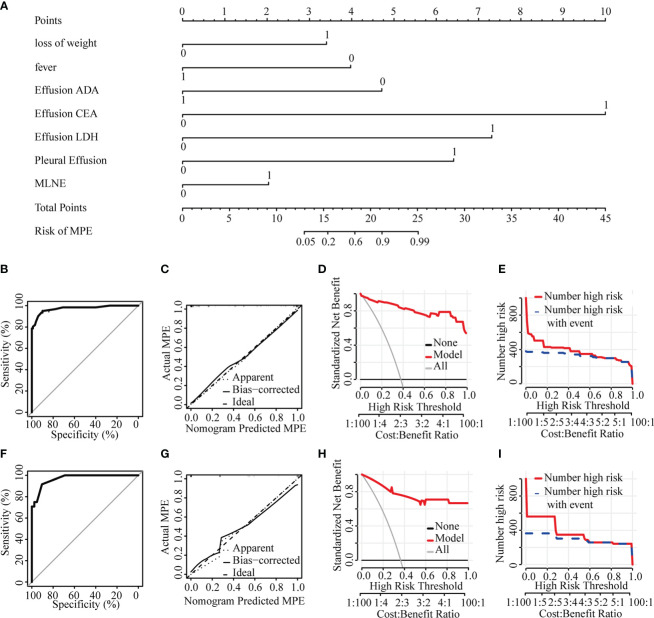
Diagnostic model of the discrimination for MPE and BPE. **(A)** Nomogram for identifying MPE from BPE. **(B)** ROC curve in the training set. **(C)** Calibration curve in the training set. **(D)** Clinical decision curve in the training set. **(E)** Clinical impact curve in the training set. **(F)** ROC curve in the validation set. **(G)** Calibration curve in the validation set. **(H)** Clinical decision curve in the validation set. **(I)** Clinical impact curve in the validation set. ADA, adenosine deaminase; CEA, carcino embryonic antigen; LDH, lactate dehydrogenase; MPE, malignant pericardial effusion; MLNE, mediastinal lymph node enlargement.

Most parameters such as heart rate, fever, weight loss, massive PE, mediastinal lymph node enlargement, pleural effusion, CEA, effusion LDH, effusion CEA, TP, ADA, effusion ADA, and effusion GLU were significantly different between MPE and BPE groups in the training set (**Supplementary Table S1**). Then we used a multivariable logistic regression model to get the optimal features, including loss of weight, fever, mediastinal lymph node enlargement, pleural effusion, effusion ADA, effusion LDH, and effusion CEA (**Figure 2**, **Table 2**). Next, a nomogram was developed to distinguish MPE from BPE based on the logistic regression model (**Figure 3A**). The ROC curve AUC of the nomogram was 0.974 (95%CI = 0.948-0.998), which had high diagnostic value (**Figure 3B**). The calibration curve showed that the predicted value of the nomogram diagnosis MPE was in good agreement with the actual observed value (**Figure 3C**). DCA showed that PE patients would benefit from the use of this nomogram model, rather than all or no treatment (**Figure 3D**). CIC analysis showed when the threshold probability was greater than 30% of the predictive scoring probability, the predictive model determines that MPE was highly matched with the actual MPE, which confirms that the predictive model had a very high clinical efficiency (**Figure 3E**).

**Table 2:** Multivariate logistic regression analysis of the clinical parameters in the training set.

Parameters	OR (95%CI)	P value
heartrate≥100	10.57 (0.81-138.69)	0.073
**loss of weight**	**30.26 (1.71-536.80)**	**0.020**
size	2.57 (0.65-10.13)	0.177
**fever**	**0.05 (0.01-0.54)**	**0.013**
**Mediastinal lymph node enlargement**	**8.99 (1.01-79.78)**	**0.049**
**Pleural Effusion**	**29.58 (1.17-745.95)**	**0.040**
TP>62.7g/L	0.35 (0.05-2.44)	0.287
ADA>9U/L	1.82 (0.27-12.44)	0.540
CEA>4.5ng/Ml	0.96 (0.09-10.79)	0.970
**Effusion ADA>18U/L**	**0.02 (0.00-0.98)**	**0.049**
Effusion GLU>5.23mmol/L	0.19 (0.02-1.59)	0.125
**Effusion LDH>1033U/L**	**164.58 (3.18-8515.92)**	**0.011**
**Effusion CEA>4.9ng/mL**	**1684.04 (21.49-131981.48)**	**0.001**
ESR>20mm/H	0.25 (0.03-2.00)	0.193

OR, odds ratio; CI, confidence interval; TP, total protein; ADA, adenosine deaminase; GLU, glucose; LDH, lactate dehydrogenase; ESR, erythrocyte sedimentation; CEA, carcino embryonic antigen. Parameters with P values less than 0.05 were shown in bold values.

The nomogram model with an AUC of 0.970 (95%CI = 0.938-1.000) showed good discrimination ability in the validation set (**Figure 3F**). The calibration curve showed that the predicted value of the nomogram was in good agreement with the actual observed value (**Figure 3G**). DCA showed that PE patients would benefit from using this nomogram model (**Figure 3H**). CIC showed the model prediction was accurate in predicting high risk MPE cases when the risk threshold was about 0.3~1.0 (**Figure 3I**).

In order to make the prediction model more suitable for doctors using in clinical work, we converted the nomogram to the scoring system: loss of weight (3 points), no fever (4 points), mediastinal lymph node enlargement (2 points), pleural effusion (6 points), effusion ADA ≦18U/L (5 points), effusion LDH >1033U/L (7 points), and effusion CEA >4.9g/mL (10 points) (**Table 3**). The optimal cut-off value for MPE diagnosis was 16 points according to the ROC curve. When the total score exceeded 16 points, PE were more likely to be diagnosed as MPE, while the total score was lower than 16 points, PE was more likely to be diagnosed as BPE. The AUC, specificity, sensitivity, PPV, NPV, PLR and NLR were 0.974, 95.1%, 91.0%, 85.6%, 96.8%, 10.56, and 0.054, respectively, in the training set (**Table 4**). When the critical point was also set to 16, AUC, specificity, sensitivity, PPV, NPV, PLR, and NLR were 0.950, 83.3%, 95.2%, 90.9%, 90.9%, 17.5, and 0.175, respectively, in the validation set (**Table 4**).

**Table 3:** Scoring system developed from a nomogram of the training set.

Parameters	Score modified from nomogram
Fever (No)	4
Loss of weight	3
Pleural Effusion (Yes)	6
Mediastinal lymph node enlargement (Yes)	2
Effusion ADA ≦18U/L	5
Effusion LDH>1033U/L	7
Effusion CEA>4.9g/mL	10

ADA, adenosine deaminase; LDH, lactate dehydrogenase; CEA, carcino embryonic antigen.

**Table 4:** Accuracy of the prediction score for differentiating MPE from BPE .

Parameters	Training set	Validation set	Lung cancer with MPE/BPE	MPE with atypical cell/BPE
AUC	0.974 (0.949-0.999)	0.950 (0.898-1.000)	0.980 (0.959-1.000)	0.940 (0.884-0.996)
Sensitivity (%)	95.1 (85.4-98.7)	83.3 (61.8-94.5)	92.2 (82.0-97.1)	85.7 (66.4-95.3)
Specificity (%)	91.0 (83.2-95.5)	95.2 (82.6-99.2)	95.1 (82.2-99.2)	92.3 (86.2-95.9)
PPV (%)	85.6 (75.5-93.3)	90.9 (69.4-98.4)	96.7 (87.7-99.4)	68.6 (50.6-82.6)
NPV (%)	96.8 (90.3-99.2)	90.9 (77.4-97.0)	88.6 (74.6-95.7)	97.0 (92.1-99.0)
PLR	10.56 (5.65-19.75)	17.50 (4.47-68.48)	18.90 (4.88-73.16)	11.06 (6.15-19.91)
NLR	0.05 (0.02-0.16)	0.18 (0.07-0.43)	0.08 (0.035-0.19)	0.16 (0.06-0.38)

MPE, malignant pericardial effect; BPE, beneficial pericardial effect; AUC, area under curve; PPV, positive predictive value; NPV, negative predictive value; PLR, positive likelihood ratio; NLR, negative likelihood ratio.

### Diagnostic significance of scoring system in differentiating lung cancer complicated with MPE from TPE

Because tuberculosis was still the main cause of pericardial effusion in developing countries, and TPE with atypical symptoms was easy to be confused with MPE cause by lung cancer in the clinical environment, we specially apply this scoring system to distinguish MPE caused by lung cancer from TPE. We found that the AUC value of the scoring system used to distinguish MPE caused by lung cancer from TPE was 0.980 (95% CI = 0.959-1.000). When the total score exceeded16, it had good specificity, sensitivity, PPV, NPV, PLR, and NLR values, as shown in **Table 4**.

### Diagnostic significance of scoring system in differentiating MPE with atypical cells from BPE

It was difficult to diagnose some atypical cells only with cytological specimens, and some auxiliary tools were often needed to achieve a clear diagnosis. Therefore, we specially apply this scoring system to distinguish MPE from BPE. We found that the AUC value of the scoring system to identify MPE with atypical cells from BPE was 0.940 (95% CI = 0.884-0.996), which had a good diagnostic performance. When the total score exceeded 16, it had good specificity, sensitivity, PPV, NPV, PLR, and NLR values (**Table 4**).

## Discussion

MPE is a common symptom of tumor invading pericardium, sometimes even the first symptom of tumor patients (12). The existence of MPE will not only seriously affect the life quality of patients but also represent the late stage of the disease. The average survival time of these patients is rarely more than 12 months (13). In addition, about 1/3 cancer patients with PE will have pericardial tamponade, resulting in hemodynamic instability and death (14). The clinical manifestations of patients with MPE lack of specificity, and distinguishing MPE from BPE may be a high challenge. Exfoliative cytology and diagnostic pericardial biopsy of pericardial effusion are of decisive significance for the diagnosis of MPE, but the sensitivity of these methods is relatively low. In this study, the sensitivity of cytology is 67.1%. Therefore, the primary goal of this study is to establish an accurate and efficient early diagnosis model of MPE.

Pericardial effusion specimens are not common, so there are few reports on the early diagnosis of MPE compared with pleural effusion or peritoneal effusion. Karatolios K et al. analyzed CEA, CA19-9, CA72-4, squamous cell carcinoma antigen (SCC), and neuron specific enolase (NSE) in the pericardial effusion of 29 patients with MPE and 25 patients with BPE, and found that measuring the level of CA 72-4 in pericardial fluid has certain diagnostic value for MPE (15). Nakamura T et al. analyzed 125 PE patients who underwent pericardiocentesis and found that low pericardial blood glucose level and high CT attenuation value had a certain suggestive effect on MPE (16). However, most biomarkers are used alone and cannot provide adequate evidence to identify MPE and BPE accurately. With the development of analytical methods, the establishment of prediction models based on multiple clinical characteristics or indicators has attracted more and more attention and application in medical research and clinical practice (17, 18).

Nomogram is to predict the probability of individual specific clinical outcomes through a certain function transformation relationship by constructing a multivariate regression model. And it transforms the complex regression equation into simple and visual graphics so that the results of the prediction model can be displayed more intuitively and has a higher use value. In this study, we collected 29 routine parameters easily obtained in the clinical practice of 227 patients with PE and established a nomogram model to distinguish MPE and BPE based on lasso regression and multiple logistic regression model. The nomogram model includes loss of weight, fever, mediastinal lymph node enlargement, pleural effusion, effusion ADA, effusion LDH, and effusion CEA.

In order to make the model more suitable for doctors to use in clinical work, we transformed the nomogram into a scoring system. Patients with a score of more than 16 are more likely to be diagnosed with MPE. We used sensitivity, specificity, PPV, NPV, PLR, and NLR to evaluate the accuracy of the scoring system and found that the scoring system has good diagnostic performance. Therefore, the scoring system is a quantitative and valuable tool which can be used to quickly distinguish MPE from BPE. Szturmowicz M et al. has developed a scoring system based on mediastinal lymph node enlargement, effusion Cyfra 21-1, effusion CEA, bloody effusion, signs of imminent cardiac tamponade and heart rate faster than 90 beats/min (19). On this basis, we have established a new scoring system combining the clinical features and effusion biochemical parameters, which has improved the diagnostic accuracy to a certain extent.

Lung cancer related MPE and TPE have many similarities in clinical characteristics and laboratory indicators. Some patients with malignant pericardial effusion cannot find tumor cells and are easy to be misdiagnosed as tuberculous pericardial effusion (20). Therefore, finding a new method to differentiate lung cancer with MPE and TPE is of great significance. We applied the scoring system to the diagnosis of these two diseases and found that the scoring system has good diagnostic performance in distinguishing MPE and TPE related to lung cancer. When the total score exceeded 16, the patient is more likely to be diagnosed with MPE.

Our study aims to design a new quantitative tool so that clinicians can predict the probability of MPE and BPE, and then help doctors choose treatment options and predict prognosis. Our scoring system is based on various common clinical and laboratory indicators. These indicators have been carried out in most hospitals, even grass-roots hospitals, and are suitable for wide clinical applications. Therefore, we recommend that the new scoring model would be widely used in most hospitals to distinguish MPE from BPE quickly.

There are still some deficiencies in this study. Firstly, this study was a single center retrospective study, which may have some bias. Secondly, only internal validation was carried out for the model, and no further external validation was carried out. Third, no other tumor markers except CEA were evaluated for pericardial effusion. Therefore, prospective studies with a larger sample size from multiple centers were needed to verify the diagnostic model.

## Conclusion

In conclusion, loss of weight, fever, mediastinal lymph node enlargement, pleural effusion, effusion ADA, effusion LDH, and effusion CEA are of great significance in distinguishing MPE from BPE. Although the scoring system developed in this study has high diagnostic value in distinguishing MPE and BPE, it still needs to be further verified in a multicenter prospective study.

## Data availability statement

The raw data supporting the conclusions of this article will be made available by the authors, without undue reservation.

## Ethics statement

The studies involving human participants were reviewed and approved by the ethics committee of Taizhou hospital, Zhejiang Province (K20220104). Written informed consent for participation was not required for this study in accordance with the national legislation and the institutional requirements.

## Author contributions

TL and GL design and conceptualized the study. XJ design and conceptualized study, analyzed the data and drafted the manuscript. MF and LH collected the data and revised the manuscript. QZ and YY collected the data. All authors contributed to the article and approved the submitted version.

## Acknowledgments

The authors thank all patients for their participation in this study.

## Conflict of interest

The authors declare that the research was conducted in the absence of any commercial or financial relationships that could be construed as a potential conflict of interest.

## Publisher’s note

All claims expressed in this article are solely those of the authors and do not necessarily represent those of their affiliated organizations, or those of the publisher, the editors and the reviewers. Any product that may be evaluated in this article, or claim that may be made by its manufacturer, is not guaranteed or endorsed by the publisher.

## Supplementary material

Supplementary Figure 1 Receiver operative characteristic (ROC) analysis were used to determine the optimal cut-offs of categorical variables. age **(A)**, effusion TP **(B)**, effusion ALB **(C)**, effusion ADA **(D)**, effusion GLU **(E)**, effusion LDH **(F)**, effusion CEA **(G)**, TP **(H)**, ALB **(I)**, ADA **(J)**, GLU **(K)**, LDH **(L)**, CEA **(M)**, hs-CRP **(N)**, ESR **(O)** and Karyocyte count **(P)**.

## Abbreviations

TP, total protein; ALB, albumin; ADA, adenosine deaminase; GLU, glucose; LDH, lactate dehydrogenase; CEA, carcinoembryonic antigen; hs-CRP, high-sensitivity C-reactive protein; ESR, erythrocyte sedimentation ratio.
