# Supplementary figures and images for: Development and validation a simple scoring system to identify malignant pericardial effusion
Source: Front Oncol. 2022 Dec 1;12:1012664. doi: 10.3389/fonc.2022.1012664 (PMC9751446; doi:10.3389/fonc.2022.1012664)

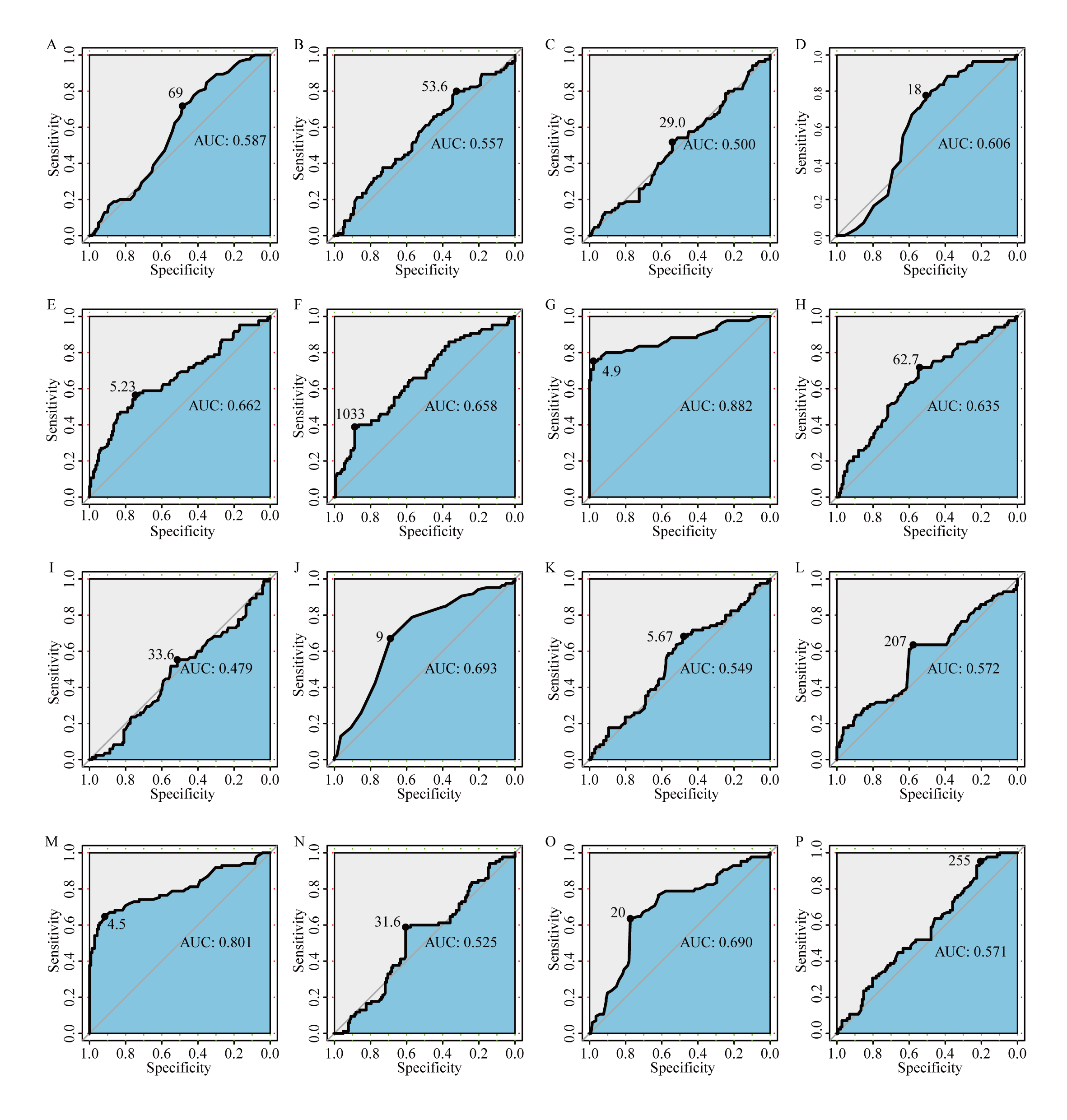

Supplement: Supplementary Figure 1 — Receiver operative characteristic (ROC) analysis were used to determine the optimal cut-offs of categorical variables. age (A), effusion TP (B), effusion ALB (C), effusion ADA (D), effusion GLU (E), effusion LDH (F), effusion CEA (G), TP (H), ALB (I), ADA (J), GLU (K), LDH (L), CEA (M), hs-CRP (N), ESR (O) and Karyocyte count (P). [file Image_1.tif]
