# Supplementary material for: Development and validation a simple scoring system to identify malignant pericardial effusion
Source: Front Oncol. 2022 Dec 1;12:1012664. doi: 10.3389/fonc.2022.1012664 (PMC9751446; doi:10.3389/fonc.2022.1012664)
Supplement: Supplementary file 2 [file Table_1.docx]

**Supplementary Table 1: Comparison of clinical parameters between MPE and BPE in the training set.**

| level | BPE (n=100) | MPE (n=61) | P value |
| --- | --- | --- | --- |
| Age (%） |  |  | 0.423 |
| ≦69 years | 22(22.0) | 10(16.4) |  |
| >69 years | 78(78.0) | 51(83.6) |  |
| Gender (%) |  |  | 0.506 |
| Male | 59 (59.0) | 40 (65.6) |  |
| Female | 41 (41.0) | 21 (34.4) |  |
| fever (%) |  |  | 0.001 |
| No | 49 (49.0) | 47 (77.0) |  |
| Yes | 51 (51.0) | 14 (23.0) |  |
| Heartrate≥100 (%) |  |  | 0.025 |
| No | 77 (77.0) | 36 (59.0) |  |
| Yes | 23 (23.0) | 25 (41.0) |  |
| Chest discomfort (%) |  |  | 0.488 |
| No | 24 (24.0) | 11 (18.0) |  |
| Yes | 76 (76.0) | 50 (82.0) |  |
| Chest pain (%) |  |  |  |
| No | 76 (76.0) | 49 (80.3) | 0.657 |
| Yes | 24 (24.0) | 12 (19.7) |  |
| Anepithymia (%) |  |  |  |
| No | 65 (65.0) | 32 (52.5) | 0.158 |
| Yes | 35 (35.0) | 29 (47.5) |  |
| Loss of weight (%) |  |  | 0.028 |
| No | 92 (92.0) | 48 (78.7) |  |
| Yes | 8 (8.0) | 13 (21.3) |  |
| Size(%) |  |  | 0.008 |
| Mild < 10 mm | 19 (19.0) | 3 (4.9) |  |
| Moderate 10-20 mm | 39 (39.0) | 19 (31.1) |  |
| Large > 20 mm | 42 (42.0) | 39 (63.9) |  |
| Onset(%) |  |  | 0.799 |
| acute (≦7 days) | 25 (25.0) | 13 (21.3) |  |
| subacute (8 days - 3 months) | 48 (48.0) | 29 (47.5) |  |
| chronic (>3 months) | 27 (27.0) | 19 (31.1) |  |
| Pleural Effusion (%) |  |  | 0.004 |
| No | 26 (26.0) | 4 (6.6) |  |
| Yes | 74 (74.0) | 57 (93.4) |  |
| [Hemopericardium](../../../../../Program Files (x86)/Youdao/Dict/8.10.5.0/resultui/html/index.html%23/javascript:;" \o "file:///D:\\Program Files (x86)\\Youdao\\Dict\\8.10.5.0\\resultui\\html\\index.html#\\javascript:;) |  |  | 0.001 |
| No | 49 (49.0) | 13 (21.3) |  |
| Yes | 51 (51.0) | 48 (78.7) |  |
| Pericardiac thickening (%) |  |  | 0.324 |
| No | 86 (86.0) | 48 (78.7) |  |
| Yes | 14 (14.0) | 13 (21.3) |  |
| Mediastinal lymph node enlargement (%) |  |  | 0.013 |
| No | 80 (80.0) | 37 (60.7) |  |
| Yes | 20 (20.0) | 24 (39.3) |  |
| hs-CRP (%) |  |  | 0.019 |
| ≦31.6 mg/L | 42 (42.0) | 38 (62.3) |  |
| >31.6 mg/L | 58 (58.0) | 23 (37.7) |  |
| ESR (%) |  |  | <0.001 |
| ≦20 mm/H | 25 (25.0) | 38 (62.3) |  |
| >20 mm/H | 75 (75.0) | 23 (37.7) |  |
| TP (%) |  |  | 0.010 |
| ≦62.7 g/L | 45 (45.0) | 41 (67.2) |  |
| >62.7 g/L | 55 (55.0) | 20 (32.8) |  |
| ALB (%) |  |  | 1.000 |
| ≦33.6 g/L | 20 (20.0) | 13 (21.3) |  |
| >33.6 g/L | 80 (80.0) | 48 (78.7) |  |
| ADA (%) |  |  | <0.001 |
| ≦9 U/L | 37 (37.0) | 46 (75.4) |  |
| >9 U/L | 63 (63.0) | 15 (24.6) |  |
| GLU (%) |  |  | 0.060 |
| ≦5.67 mmol/L | 56 (56.0) | 44 (72.1) |  |
| >5.67 mmol/L | 44 (44.0) | 17 (27.9) |  |
| LDH (%) |  |  | 0.011 |
| ≦207 U/L | 53 (53.0) | 19 (31.1) |  |
| >207 U/L | 47 (47.0) | 42 (68.9) |  |
| CEA (%) |  |  | <0.001 |
| ≦4.5 ng/mL | 90 (90.0) | 19 (31.1) |  |
| >4.5 ng/mL | 10 (10.0) | 42 (68.9) |  |
| Karyocyte count (%) |  |  | 0.015 |
| ≦255*106/L | 20 (20.0) | 3 (4.9) |  |
| >255*106/L | 80 (80.0) | 58 (95.1) |  |
| Effusion TP (%) |  |  | 0.455 |
| ≦53.6 g/L | 50 (50.0) | 35 (57.4) |  |
| >53.6 g/L | 50 (50.0) | 26 (42.6) |  |
| Effusion ALB (%) |  |  | 0.616 |
| ≦29.0 g/L | 52 (52.0) | 35 (57.4) |  |
| >29.0 g/L | 48 (48.0) | 26 (42.6) |  |
| Effusion ADA (%) |  |  | 0.014 |
| ≦18 U/L | 53 (53.0) | 45 (73.8) |  |
| >18 U/L | 47 (47.0) | 16 (26.2) |  |
| Effusion GLU (%) |  |  | <0.001 |
| ≦5.23 mmol/L | 24 (24.0) | 36 (59.0) |  |
| >5.23 mmol/L | 76 (76.0) | 25 (41.0) |  |
| Effusion LDH (%) |  |  | <0.001 |
| ≦1033 U/L | 87 (87.0) | 31 (50.8) |  |
| >1033 U/L | 13 (13.0) | 30 (49.2) |  |
| Effusion CEA (%) |  |  | <0.001 |
| ≦4.9 ng/mL | 97 (97.0) | 13 (21.3) |  |
| >4.9 ng/mL | 3 (3.0) | 48 (78.7) |  |

Abbreviations: BPE, benign pericardial effusion; MPE, malignant pericardial effusion; hs-CRP, high-sensitivity C-reactive protein; TP, total protein; ALB, albumin; ADA, adenosine deaminase; GLU, glucose; LDH, lactate dehydrogenase; ESR, erythrocyte sedimentation; CEA, carcino embryonic antigen.
